# Supplementary material for: Unraveling the pathway of copper delivery to cytochrome c oxidases in the free-living bacterium Caulobacter vibrioides
Source: J Biol Chem. 2025 Oct 6;301(11):110786. doi: 10.1016/j.jbc.2025.110786 (PMC12617631; doi:10.1016/j.jbc.2025.110786)
Supplement: Supporting Data-Tables [file mmc1.pdf]

Table S1. Candidates from the genetic screen that were found to be required for *aa_3*-Cox activity in *C. vibrioides*.

Category	Candidates	Locus tag	Tn5 insertions
aa3-Cox	CoxA	CCNA_03518	7
	CoxB	CCNA_03517	7
	CoxC	CCNA_03513	1
	hp	CCNA_03515	3
bc1 complex	ubiquinol-cytochrome *c* reductase iron-sulfur subunit	CCNA_00505	2
	ubiquinol cytochrome *c* reductase *b* subunit	CCNA_00506	11
	ubiquinol-cytochrome *c* reductase cytochrome *c1* subunit	CCNA_00507	4
Cytochrome *c* maturation (Ccm)	Cytochrome *c*-type biogenesis protein (CcmH)	CCNA_02847	1
	Cytochrome *c*-type biogenesis heme chaperone (CcmE)	CCNA_02849	2
	Heme exporter protein C (CcmC)	CCNA_03786	1
	Heme exporter protein A (CcmA)	CCNA_03782	1
	disulfide interchange protein (CcmG)	CCNA_03788	2
	Cytochrome *c*	CCNA_03030	2
Heme A	Heme A synthase CtaA	CCNA_01438	1
Thiol	disulfide interchange protein TlpA	CCNA_02293	2
Chaperones	PccA	CCNA_03617	1
	CtaG	CCNA_03514	2
Transporter	TccA	CCNA_03615	7

Table S2. Bacterial strains and plasmids used in this study

Strains	Genotype and/or phenotype	Reference or source
Caulobacter vibrioides		
WT	Wild-type NA1000, synchronizable variant of CB15	(37)
Δ*fixI*	Knock-out strain for *fixI* gene (CCNA_01473)	This study
Δcbb_3	Knock-out strain for the *ccoN*, *ccoO*, *ccoQ*, *ccoP* genes (CCNA_01467) (CCNA_01468) (CCNA_01469) (CCNA_01470)	This study
Δaa_3	Knock-out strain for the *coxB* and *coxA* genes (CCNA_03517), (CCNA_03518)	This study
Δaa_3Δcbb_3	Double knock-out strain of both aa_3 and cbb_3 genes, as mentioned earlier	This study
Δ*fixI*Δcbb_3	Double knock-out strain of both *fixI* and cbb_3 genes	This study
Δ*fixI*Δaa_3	Double knock-out strain of both *fixI* and aa_3 genes	This study
Δ*sco1*	Knock-out strain for *sco1* gene (CCNA_03618)	This study
Δ*sco2*	Knock-out strain for *sco2* gene (CCNA_00249)	This study
Δ*sco1*Δ*sco2*	Double knock-out strain of both *sco1* and *sco2* genes	This study
Δ*pccA*	Knock-out strain for *pccA* gene (CCNA_03617)	This study
Δ*pccA*Δ*sco1*Δ*sco2*	Triple knock-out strain of the three genes *pccA*, *sco1* and *sco2*	This study
Δ*pccA*Δaa_3	Double knock-out strain of both *pccA* and aa_3 genes	This study
Δ*pccA*Δcbb_3	Double knock-out strain of both *pccA* and cbb_3 genes	This study
fixI: C398A-C400A	WT strain with the two-point C398A and C400A substitutions in the chromosomal *fixI*	This study
Δ*fixI*+p*fixI*	Knock-out strain for *fixI* gene carrying a copy of *fixI* on the pMR10 under the control of the plac promoter; KanR	This study
Δ*pccA*+p*pccA*	Knock-out strain for *pccA* gene carrying a copy of *pccA* on the pMR10 under the control of the lac promoter; KanR	This study
Δ*fixI*Δcbb_3,*tccA::Tn5*	Double knock-out strain of both *fixI* and cbb_3 genes with a mini-Tn5 transposon inserted in the CCNA_03615 gene	This study
Δ*fixI*Δcbb_3,*tccA::Tn5*+p*tccA*	Double knock-out strain of both *fixI* and cbb_3 genes with a mini-Tn5 transposon inserted in the CCNA_03615 gene carrying a copy of *tccA* on the pMR20 under the control of the plac promoter; TetR	This study
Δ*fixI*Δcbb_3,*ctaG::Tn5*	Double knock-out strain of both *fixI* and cbb_3 genes with a mini-Tn5 transposon inserted in the CCNA_03514 gene	This study
Δ*fixI*Δcbb_3,*ctaG::Tn5*+p*ctaG*	Double knock-out strain of both *fixI* and cbb_3 genes with a mini-Tn5 transposon inserted in the CCNA_03514 gene carrying a copy of *ctaG* on the pMR20 under the control of the plac promoter; TetR	This study
Δ*zctP*	Knock-out strain for *zctP* gene (CCNA_02811)	This study
Δ*zctP*Δ*fixI*	Double Knock-out strain for both *fixI* and *zctP* genes	This study
Δ*zctP*+p*zctP*	Knock-out strain for *zctP* gene carrying a copy of *zctP* on the pMR10 under the control of the lac promoter; KanR	This study
WT+ p*zctP*	Knock-out strain carrying a copy of *zctP* on the pMR10 under the control of the lac promoter; KanR	This study
**Plasmids**		
pNTPS	mobRP4+ ori-R6K sacB; integrative vector in *C. vibrioides* for in-frame deletions; KanR	
pMR10	Low copy and replicative vector in *C. vibrioides*; KanR	
pMR20	Derivative of pMR10; TetR	

Table S3. Primers used in this study

Primer	Target	Sequence
Pr1	Δ*fixI* (Upstream *fixI* FWD)	AATGACGATCGCCGCGCAAT
Pr2	Δ*fixI* (Upstream *fixI* overlap region RWD)	CGAACATCGCCGCCgATCTTGCTGATGCAGCCGGCGC
Pr3	Δ*fixI* (Downstream *fixI* overlap region FWD)	GGCTGCATCAGCAAGATcGGCGGCGATGTTCGGAC
Pr4	Δ*fixI* (Downstream *fixI* RWD)	GGCTTGATGTCATTGCCGACA
Pr5	Δ*cbb3* (Upstream *ccoN* FWD)	GCATCTGCGCTGAACCGCCT
Pr6	Δ*cbb3* (Upstream *ccoN* overlap region RWD)	GCGTGGGCATGGGCCACGGACTCCCTGGGGCGAA
Pr7	Δ*cbb3* (Downstream *ccoP* overlap region FWD)	CCCCAGGGAGTCCGTGGCCCATGCCCACGCTCATC
Pr8	Δ*cbb3* (Downstream *ccoP* FWD)	CAGGCGCATCCGGGCGTTA
Pr9	Δ*aa3* (Upstream *coxB* FWD)	CATATCGATGTCCCTCGTGA
Pr10	Δ*aa3* (Upstream *coxB* overlap region RWD)	GGACGACGGTTCACCATCCCTCACGG
Pr11	Δ*aa3* (Downstream *coxA* overlap region FWD)	CGGGCCGTGAGGGATGGTGAACCGTC
Pr12	Δ*aa3* (Downstream *coxA* overlap region RWD)	GGCCAGCCAATTGACCGCAA
Pr13	Δ*sco1* (Upstream *sco1* FWD)	CCTGCGCCGGCTGATCACGC
Pr14	Δ*sco1* (Upstream *sco1* overlap region RWD)	AAGGATTTGCCCGGGCAGTGATGCATCCCCGACAT
Pr15	Δ*sco1* (Downstream *sco1* overlap region FWD)	GGGGATGCATCACTGCCCGGGCAAATCCTTTCCCG
Pr16	Δ*sco1* (Downstream *sco1* RWD)	GGGATCTGCGTGCGCGCGT
Pr18	Δ*sco2* (Upstream *sco2* FWD)	CAGGGTGGCCGCTGCGAGGG
Pr19	Δ*sco2* (Upstream *sco2* overlap region RWD)	AATTTTTACGCCGGAGCGCGGTTCATCCTTTTACT
Pr20	Δ*sco2* (Downstream *sco2* overlap region FWD)	AAGGATGAACCGCGCTCCGGCGTAAAAATTCGCGA
Pr21	Δ*sco2* (Downstream *sco2* RWD)	GATGAAGACGGCGTGGTCGG
Pr22	Δ*pccA* (Upstream *pccA* FWD)	TGACAGGCGGCTTCACCCTG
Pr23	Δ*pccA* (Upstream *pccA* overlap region RWD)	GAGCGATGGCCCGCACTTCAGGCTCCGGTCTGTGC
Pr24	Δ*pccA* (Downstream *pccA* overlap region FWD)	GACCGGAGCCTGAAGTGCGGGCCATCGCTCTGAC
Pr25	Δ*pccA* (Downstream *pccA* RWD)	CCCCTTGGCGTCGAACAGAT

Primer	Target	Sequence
Pr26	*fixI*: C398A-C400A (Upstream region FWD)	GGACAACGCCCTGATCACCG
Pr27	*fixI*: C398A-C400A (Upstream overlap region RWD)	ACCGCCAGGCCCAGGGCTGCCGGTGCAGTCACGAT
Pr28	*fixI*: C398A-C400A (Downstream overlap region FWD)	TGACTGCACCGGCAGCCCTGGGCCTGGCGGTGC
Pr29	*fixI*: C398A-C400A (Downstream region RWD)	CAGCCGTCTCGACGCAGTCCT
Pr30	Δ*zctP* (Upstream *zctP* FWD)	TGTTCGTCCTGCCGGCGAT
Pr31	Δ*zctP* (Upstream *zctP* overlap region RWD)	GCGCGAGGTCGTTCGCGGCGCGTCTCCAGAGTTAG
Pr32	Δ*zctP* (Downstream *zctP* overlap region FWD)	AACTCTGGAGACGCGCCGCGAACGACCTCGCGCCAA
Pr33	Δ*zctP* (Downstream *zctP* RWD)	TCGTTCCTTCGTCACTTCGATG
Pr34	Forward amplification of *zctP* (CCNA_02811) gene with restriction enzyme Xbal	tgctctagaATGGCCGCCGGCATAAAGCCC
P35	Reverse amplification of *zctP* (CCNA_02811) gene with restriction enzyme KpnI	ggtaccTTCGCTCACGCCGTCCGGTC
Pr36	Forward amplification of the *pccA* gene CCNA_03617 with restriction enzyme HindIII	CCCAAGCTTATGAAGACCCTGACCCTGCT
Pr37	Reverse amplification of the *pccA* gene CCNA_03617 with restriction enzyme XbaI	TGCTCTAGATCAGTGATGCATCCCCGACA
Pr38	Forward amplification of the *fixI* gene CCNA_01473 with restriction enzyme SacI	CCGAGCTCATGAGCCACAGCCTCGCC
Pr39	Reverse amplification of the *fixI* gene CCNA_01473 with restriction enzyme XbaI	TGCTCTAGATCATCGGTTGAAACTCCG
Pr40	Forward amplification of the *tccA* gene CCNA_03615 with restriction enzyme XbaI	TGCTCTAGAATGAAGTCTCTTCTGCTG
Pr41	Reverse amplification of the *tccA* gene CCNA_03615 with restriction enzyme EcoRI	CCGGAATTCTTAGAAGCTGACGGTCA
Pr42	Forward amplification of the *ctaG* gene CCNA_03513 with restriction enzyme HindIII	CCCAAGCTTATGTCGCAAAACCCCAC
Pr43	Reverse amplification of the *ctaG* gene CCNA_03513 with restriction enzyme EcoRI	CCGGAATTCCTATAGACCTCTCGACGG

Table S4. p-values. A t-test was performed using GraphPad Prism.

**Fig. 1D Unpaired t-test**	**Summary**	**Adjusted P Value**
WT vs. Δ*fixI*	ns	0.3452
**Fig. 2D Unpaired t-test**		
WT vs. Δ*fixI* (CoxA)	ns	0.3503
WT vs. Δ*fixI* (CoxB)	ns	0.6520
WT vs. Δ*fixI* (CoxC)	ns	0.6240
WT vs. Δ*fixI* (CcoN)	ns	0.3118
WT vs. Δ*fixI* (CcoP)	ns	1
WT vs. Δ*fixI* (CcoO)	ns	0.3552
WT vs. Δ*fixI* (CcoG)	ns	0.08
**Fig. 3B Unpaired t-test**		
WT vs. Δ*pccA* (CoxA)	ns	0.1356
WT vs. Δ*pccA* (CoxB)	**	0.0043
WT vs. Δ*pccA* (CcoN)	ns	0.3118
WT vs. Δ*pccA* (CcoP)	ns	0.3046
WT vs. Δ*pccA* (CcoO)	ns	0.3349
WT vs. Δ*pccA* (CcoS)	ns	0.6779
**Fig. 4C Unpaired t-test**		
WT vs. Δ*fixIcbb3,tccA::Tn5* (10 mM Fe)	ns	0.7889
WT vs. Δ*fixIcbb3,tccA::Tn5* (2.5 mM Fe)	ns	0.05125
WT vs. Δ*fixIcbb3,tccA::Tn5* (15 µM Cu)	ns	0.70344
**Fig. 4E Unpaired t-test**		
WT vs. Δ*aa3* (TccA CCNA_03615)	***	0.0101
WT vs. Δ*aa3* (PccA CCNA_03617)	***	0.017
WT vs. Δ*aa3* (Sco1 CCNA_03618)	***	0.0039

**Fig. 5A Unpaired t-test**	**Summary**	**Adjusted P Value**
WT vs. Δ*fixI*	ns	0.5283
WT vs. Δ*cbb3*	ns	0.1740
WT vs. Δ*aa3*	**	0.0062
WT vs. Δ*aa3*Δ*cbb3*	**	0.0039
WT vs. Δ*fixI*Δ*aa3*	*	0.019
WT vs. Δ*fixI*Δ*cbb3*	ns	0.4942
**Fig. 5B Unpaired t-test**		
WT vs. Δ*sco1*	ns	0.409
WT vs. Δ*sco2*	ns	0.658
WT vs. Δ*sco1*Δ*sco2*	ns	0.5
WT vs. Δ*pccA*	**	0.0023
WT vs. Δ*pccA*Δ*sco1*Δ*sco2*	**	0.0052
**Fig. 5B Unpaired t-test**		
Δ*fixI*Δ*cbb3* vs. Δ*fixI*Δ*cbb3,tccA::Tn5*	**	0.0045
Δ*fixI*Δ*cbb3* vs. Δ*fixI*Δ*cbb3,ctaG::Tn5*	***	0.0004
**Fig. 5D Unpaired t-test**		
Δ*cbb3* vs. Δ*aa3* (+O2)	ns	0.1454
Δ*cbb3* vs. Δ*aa3* (-O2)	**	0.0013
**Fig. 5E Unpaired t-test**		
CcoN (+O2 vs. -O2)	**	0.0014
CcoP (+O2 vs. -O2)	***	0.0003
CcoO (+O2 vs. -O2)	**	0.0012
CcoI (+O2 vs. -O2)	****	0.0001
CcoG (+O2 vs. -O2)	****	0.0001

**Fig. 5F Unpaired t-test**	**Summary**	**Adjusted P Value**
CoxA (+O2 vs. -O2)	ns	0.7900
CoxB (+O2 vs. -O2)	ns	0.2336
CoxC (+O2 vs. -O2)	ns	0.1338
CoxE (+O2 vs. -O2)	ns	0.2508
**Fig. S4B Unpaired t-test**		
WT vs. Δ*zctP* (PYE)	ns	0.46
WT vs. Δ*zctP* (Zn)	ns	0.34
**Fig. S5C Unpaired t-test**		
WT vs. Δ*aa3*Δ*cbb3* (qoxA)	ns	0.15
WT vs. Δ*aa3*Δ*cbb3* (qoxB)	ns	0.47
WT vs. Δ*aa3*Δ*cbb3* (Surf1)	ns	0.37
WT vs. Δ*aa3*Δ*cbb3* (CydA)	ns	0.41
WT vs. Δ*aa3*Δ*cbb3* (CydB)	ns	0.07
WT vs. Δ*aa3*Δ*cbb3* (CydC)	ns	0.44
WT vs. Δ*aa3*Δ*cbb3* (CydD)	*	0.04
