# Supplementary material for: Unraveling the pathway of copper delivery to cytochrome c oxidases in the free-living bacterium Caulobacter vibrioides
Source: J Biol Chem. 2025 Oct 6;301(11):110786. doi: 10.1016/j.jbc.2025.110786 (PMC12617631; doi:10.1016/j.jbc.2025.110786)
Supplement: Supporting Figures [file mmc2.pdf]

# Figure S1

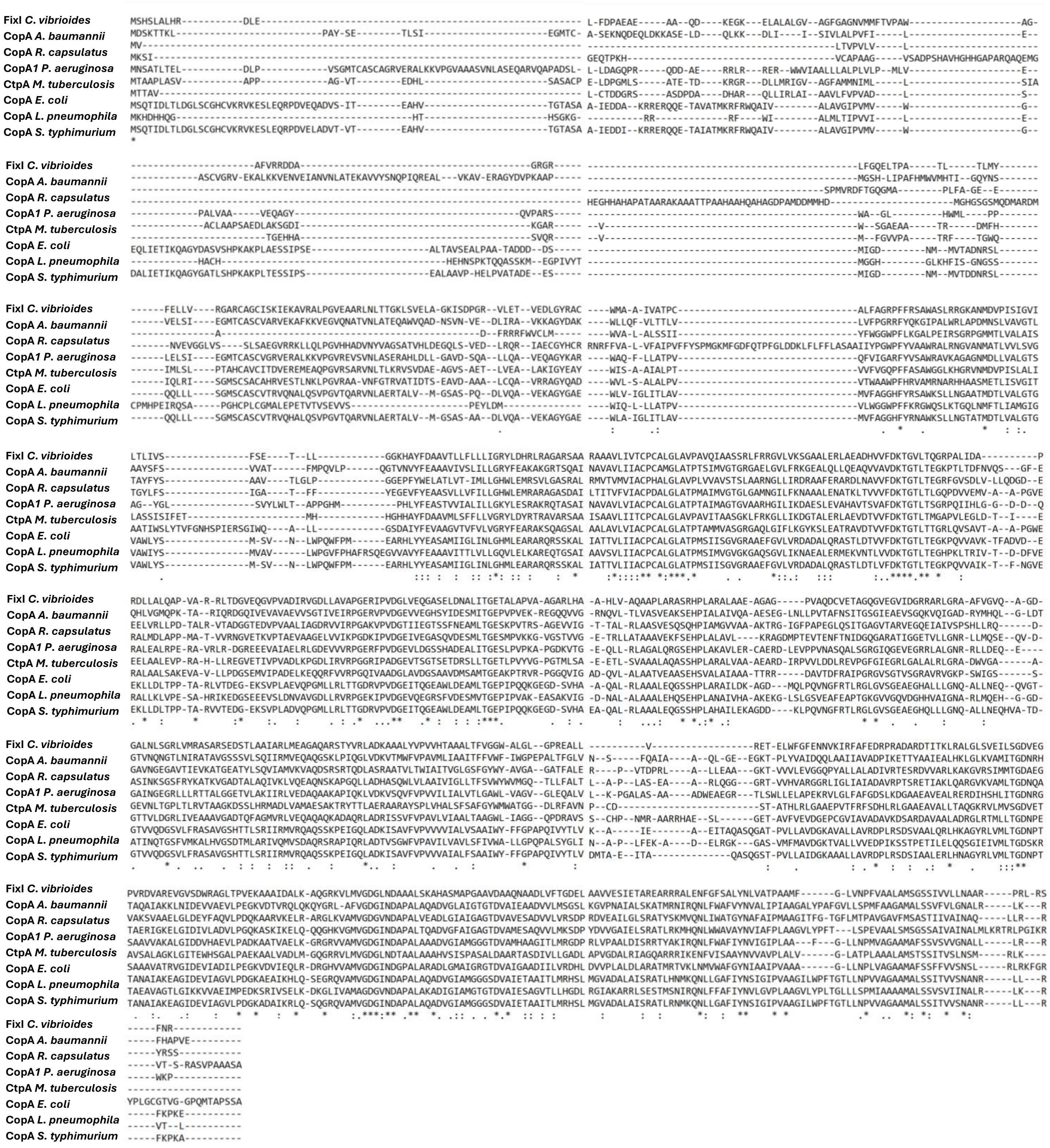

**Figure S1. Multiple sequence alignment of FixI from *C. vibrioides* and homologous P1B-type ATPases from diverse bacterial Species.** Whole amino acid sequence alignment of FixI from *C. vibrioides* with its homologs CopA from *Acinetobacter baumannii*, CopA from *Rhodobacter capsulatus*, CopA *Pseudomonas aeruginosa*, CtpA *Mycobacterium tuberculosis,* CopA *Escherichia coli*, CopA *Legionella pneumophila,* and CopA *Salmonella typhimurium*.

# Figure S2

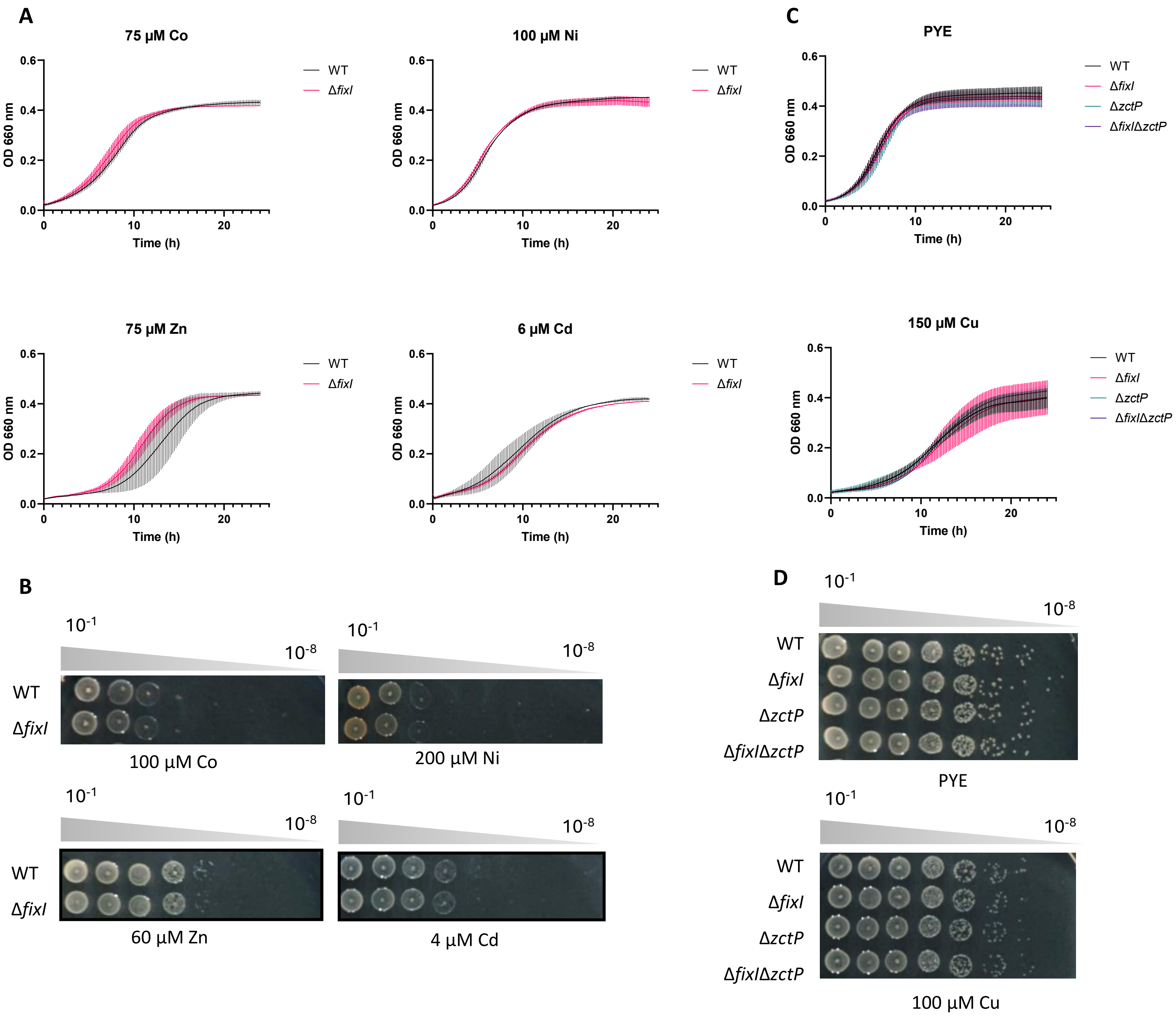


**Figure S2.** ***C. vibrioides*** **lacks the P1B-type ATPase required for Cu detoxification. A.** Growth profiles at $OD_{660}$ of WT and Δ*fixI* under Cobalt (Co), Nickel (Ni), Zinc (Zn), and Cadmium (Cd) excess. Mean ± SD, at least three biological replicates. **B**. Viability assay on rich (PYE) under Cobalt (Co), Nickel (Ni), Zinc (Zn), and Cadmium (Cd) excess. Plates were incubated for 48 h at 30 °C before imaging. Biological replicates = 3. **C.** Growth profiles at an absorbance of 660 nm of WT, Δ*fixI*, Δ*zctP,* and Δ*fixI*Δ*zctP* in PYE and moderate Cu stress, Mean ± SD, at least three biological replicates. **D.** Viability assay on rich (PYE) under excess Cu. Plates were incubated for 48 h at 30 °C before imaging. Biological replicates = 3. The WT, Δ*fixI* have been reused from Fig. 1C because all the strains from Fig. S2D originate from the same plate as the strains from Fig. 1C.

# Figure S3

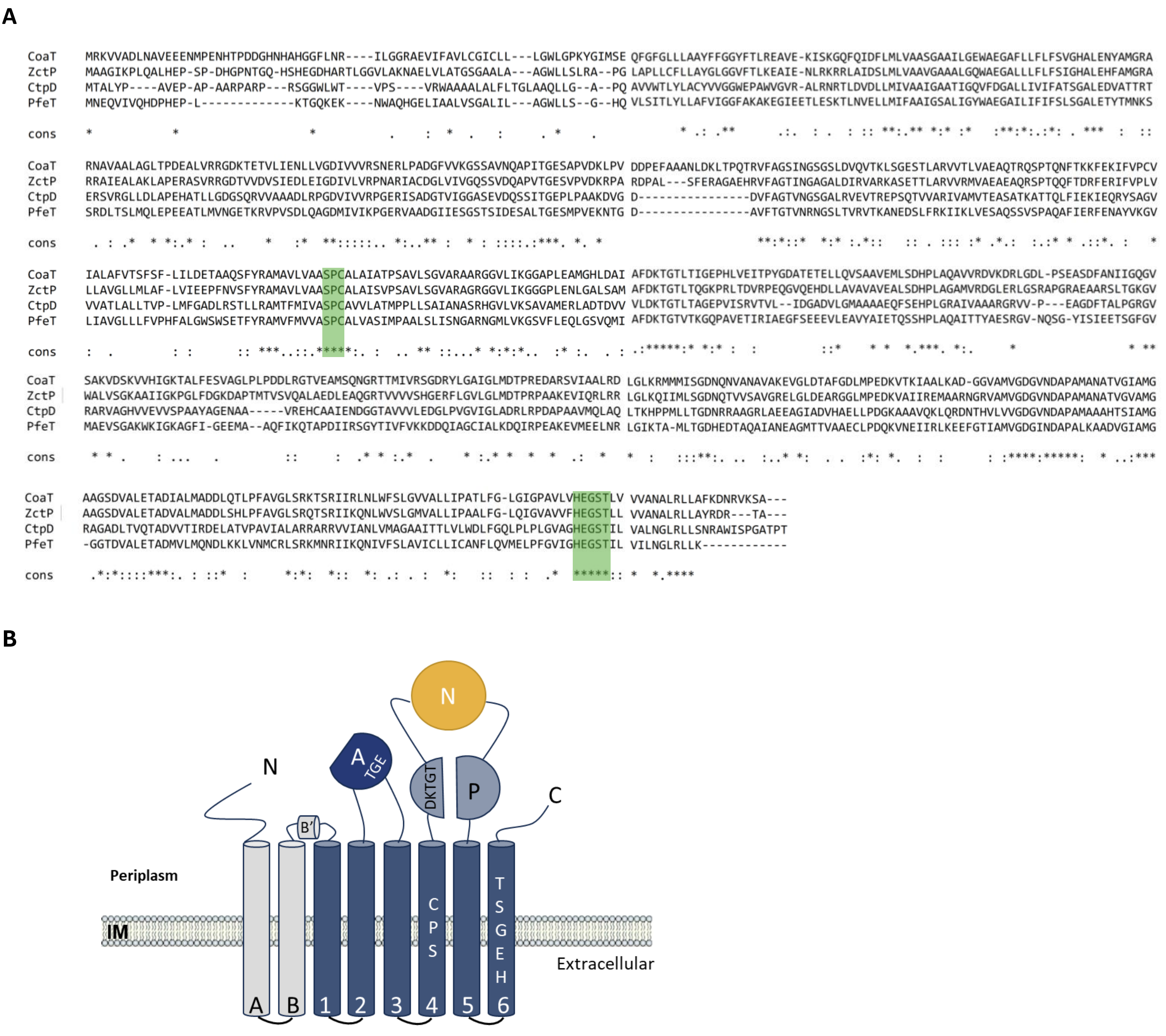


**Figure S3. ZctP seems to belong to the P1B4 subclass of P-type ATPases. A.** Whole amino acid sequence alignment of ZctP *C. vibrioides* with its homologs CoaT *Sulfitobacter sp*. NAS14-1, CtpD *Mycobacterium smegmatis* and PfeT *Bacillus subtilis*. **B.** Schematic topology of P-type ATPases showing features unique to PIB4-ATPase of ZctP. Key residues for PIB4-ATPases are highlighted.

# Figure S4

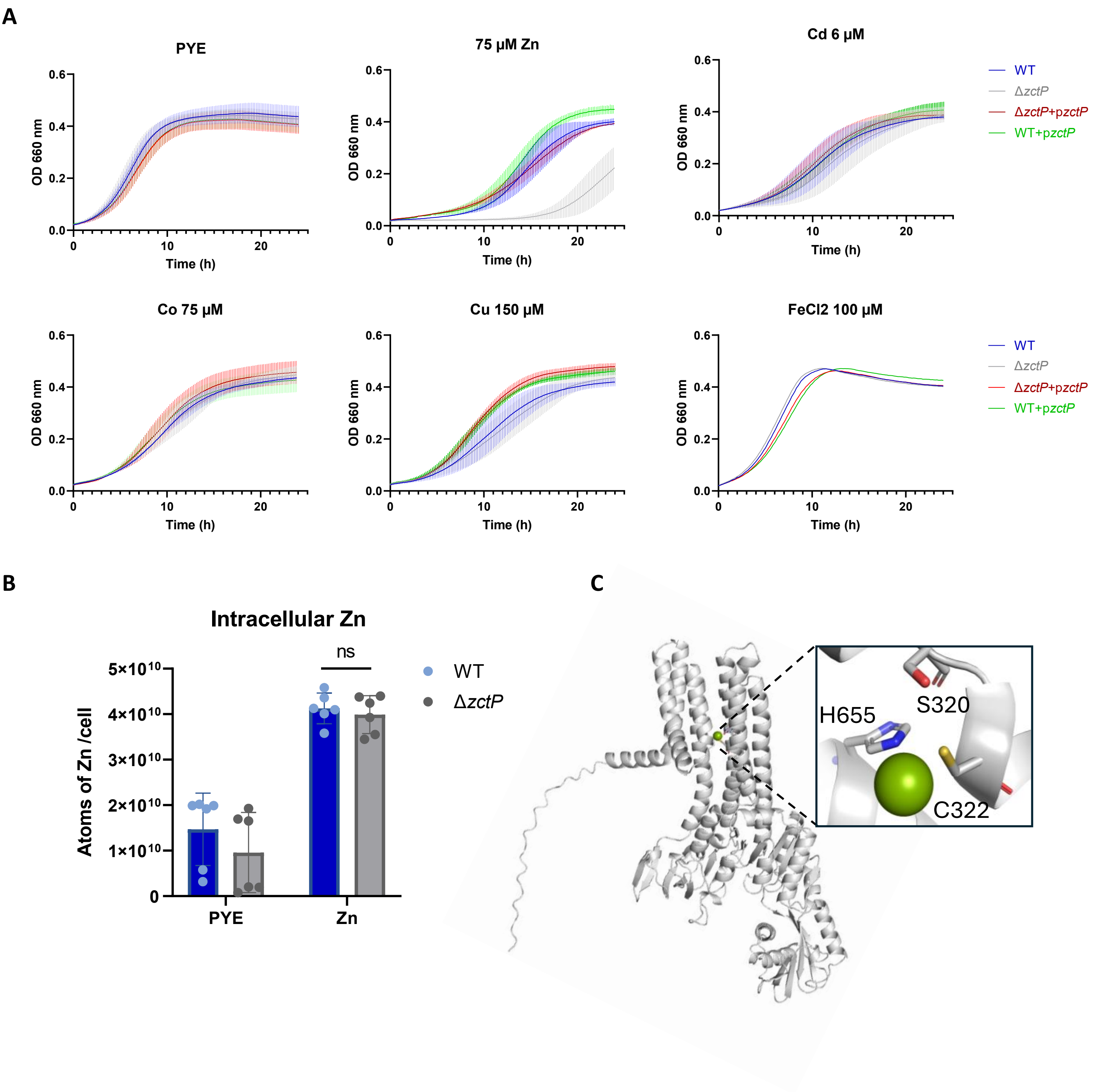


**Figure S4. ZctP is specifically required for Zn homeostasis. A.** Growth profiles at $OD_{660}$ of WT, Δ*zctP, complemented strain (*Δ*zctP*+ p*zctP) and overexpressed strain (*WT+ p*zctP)* under excess Cobalt (Co), Nickel (Ni), Zinc (Zn), and Cadmium (Cd), Cobalt (Co), Cu (Cu) and Iron (Fe) excess. Mean ± SD, at least three biological replicates except the replicate with excess Fe. **B.** Prediction by Alphafold of ZctP 3D structure with a potential Zn binding site. The model was visualized using Pymol. **C**. The Number of Cu atoms per cell of the WT and ΔzctP strains in the control condition (PYE) was exposed to $ZnSO_4$ excess for 30 min. Mean ± SD, at least three biological replicates. P-values were calculated using a t-test (**p* < 0.05, ** *p* < 0.01, *** *p* < 0.001, and ****$p$ < 0.0001) **(Table S4)**.

# Figure S5

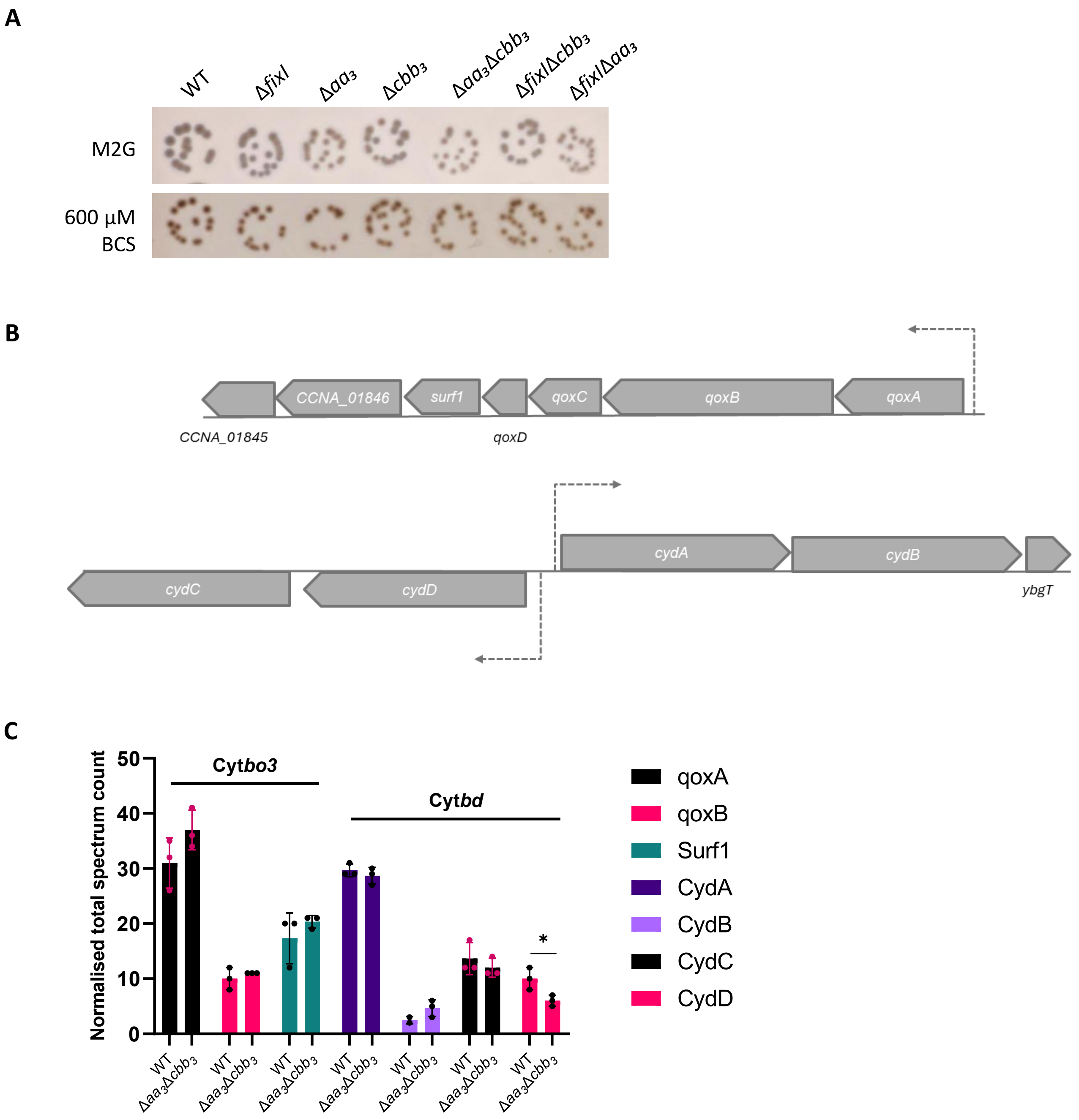


**Figure S5. *C. vibrioides* possesses two quinol oxidases: cytochrome *bd*-type quinol oxidase (Cyt*bd*) and cytochrome *bo3* quinol oxidase (Cyt*bo3*). A.** Growth and NADI phenotypes of colonies of *C. vibrioides* wild-type (WT) and mutant strains. Cells were grown aerobically at 30°C on a minimal medium (M2G) and M2G supplemented with 600 µM BCS (Cu chelator), and the presence of Cox activity was visualized by NADI staining (see Materials and Methods). **B.** Genomic organization of the Cyt*bo3* and Cyt*bd* operon, respectively. **C.** Normalized total spectrum count of peptides from Cyt*bo3* and Cyt*bd* subunits in the WT and $\Delta aa_3\Delta cbb_3$ strains grown in PYE medium, as measured by LC-MS. Individual values and means are shown. **C.** Normalized total spectrum count of peptides from Cyt*bo3* and Cyt*bd* subunits in the WT and $\Delta aa_3\Delta cbb_3$ strains grown in PYE medium, as measured by LC-MS. Individual values and means are shown. P-values were calculated using a t-test (*$p < 0.05$, ** $p < 0.01$, *** $p < 0.001$, and ****$p < 0.0001$) **(Table S4)**.

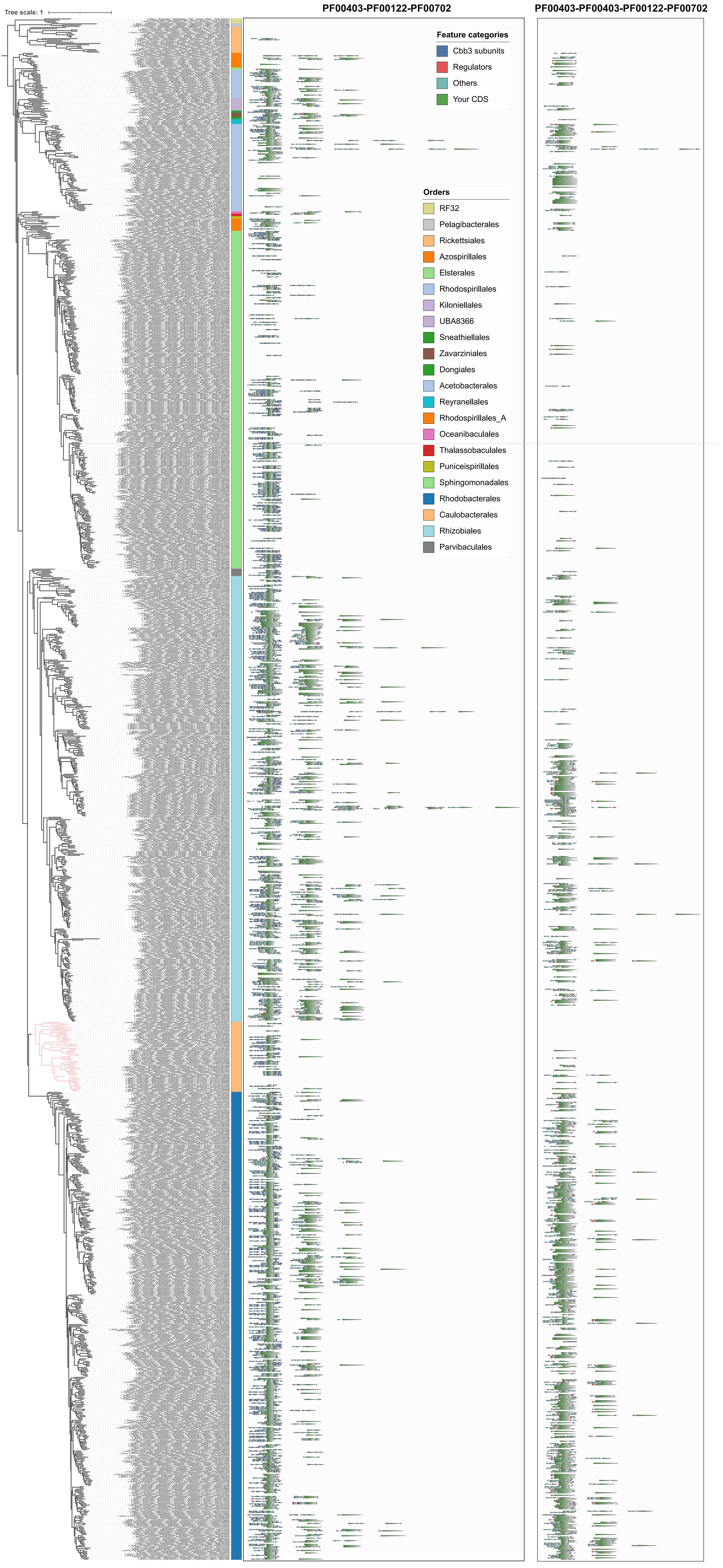
Tree scale: 1
PF00403-PF00122-PF00702
PF00403-PF00403-PF00122-PF00702
Feature categories
Cbb3 subunits
Regulators
Others
Your CDS
Orders
RF32
Pelagibacterales
Rickettsiales
Azospirillales
Elsterales
Rhodospirillales
Kiloniellales
UBA8366
Sneathiellales
Zavarziniales
Dongiales
Acetobacterales
Reyranellales
Rhodospirillales_A
Oceanibaculales
Thalassobaculales
Puniceispirillales
Sphingomonadales
Rhodobacterales
Caulobacterales
Rhizobiales
Parvibaculales

# Figure S6

**Figure S6. Genomic context of P-type ATPases across Alphaproteobacteria**. GTDB phylogenetic tree of 1,255 Alphaproteobacterial genomes, with taxonomic orders color-coded as indicated. To the right, the genomic context is shown for all proteins containing the two domain architectures of interest: PF00403–PF00122–PF00702 (left panel) and PF00403–PF00403–PF00122–PF00702 (right panel). Genes are colored by predicted function: $cbb_3$-type cytochrome c oxidase subunits (blue), regulatory elements (red), other genes (gray), and the ATPase of interest (green)

# Figure S7

**A**

```
PccA C. vibrioides    MKTLTL-LGVCAAALALTAAAPAPKV------TAADAWCRPAPTGAMAGGCYVTLT-A-GVDDRLVAVETTAAARGEIHT
PccA R. capsulatus    MIKGFA-APLCALMLALMPVSGHAHEAQAGALTIIHPSIPAVMPGAQAAAGYFTIVNAGDSADRLTAVEVDFAAMAMLHK
PCuAC R. sphaeroides  MTPFQAILAAAAVTFALPAFAD-SEL------AVTDAYARVASPAAQSGAIFMVIENHGASDDRLVSAATDAAARVELHT
                      *         .*  :** . :  ..       :  ..    . ..* :.. :..:       ***.:. .  **   :*.

PccA C. vibrioides    MSM-DGGVMRMRKLADGLALPAGKAVALKPGADHIMVIGPKIALTEGAQVPLTLKFQKAKPVKVTAVVKMPPK-P---AM
PccA R. capsulatus    TETDAAGVARMLDV-DAVEVPAGATVTFAPGGLHVMFMGLSAPIAEGVMLPGTLVFEHAGKVPVEFMVTTAGKAP---DM
PCuAC R. sphaeroides  HLAGQDGVMQMVEVKEGFPVPAHGSHALARGGDHVMLMGLTKPLKEGDEIALTLTFESGKVLEVAAPVDTSREAPMGDHM
                            ** :* .: :.. :**  : ::  *. *:*.:* . .: **  :. ** *: .  : *   *  . : *    *

PccA C. vibrioides    SGMH--H
PccA R. capsulatus    SGHAMP-
PCuAC R. sphaeroides  KGMTHGN
                      .*
```

**B**

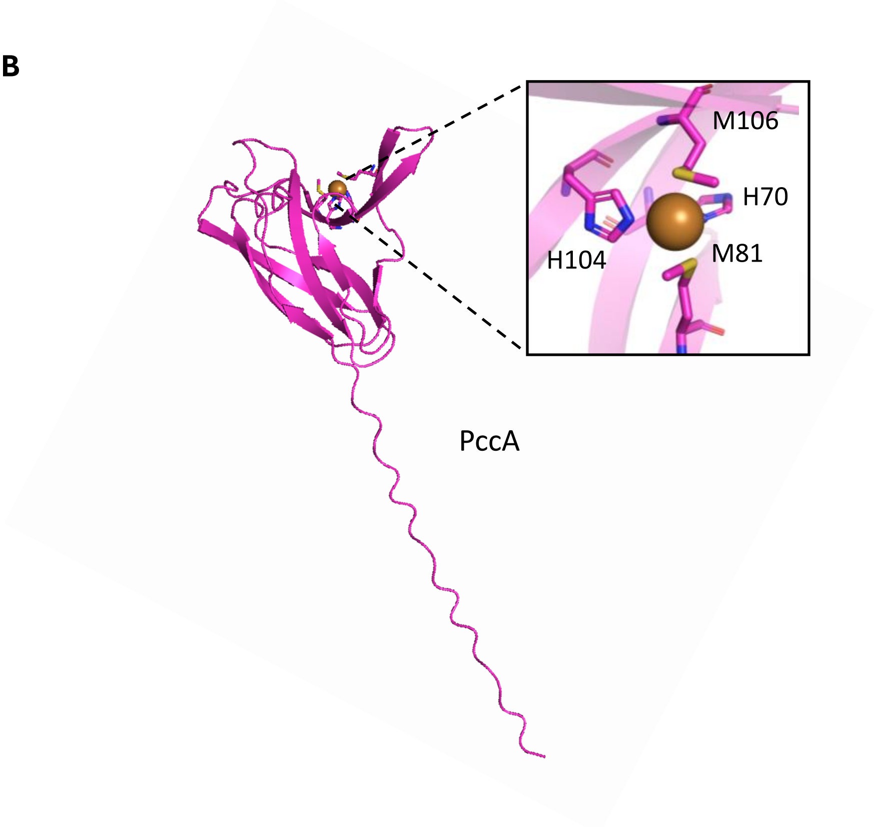


**Figure S7. PccA from *C. vibrioides* belongs to the PCuAC family. A.** The conserved H(M)$X_{10}$M$X_{21}$HXM motif from PccA sequence alignments from *C. vibrioides*, *R. capsulatus,* and *R. sphaeroides*. **B.** Alphafold predicted structure of *C. vibrioides* PccA. PccA exhibits a cupredoxin-like fold, a structural motif common in Cu-binding proteins. This fold involves two histidine and two methionine residues that could coordinate a Cu(I) ion (His 70, Met 81, His 104, and Met 106).

# Figure S8

**A**

```
Sco1 C. vibrioides  ----------------------------MPRHRLVLILACVVGLAV----------------------------AVGLAW
Sco2 C. vibrioides  ------------------------------------------MRAIALTLALLAATPLAGCGEKAAQDAAGAVIKIG---
SenC R. capsulatus  MTKLYAGVAAAAIAALLAGSAAWVFLGRSEERFAGCGANQVAGGA-----------------------------------
PrrC R. sphaeroides -------------------------------------MNVSSKTA------ALAATAAVVVVVGISAAVTLVPHETDRFA
                                                                *

Sco1 C. vibrioides  NVGVFRSEPAVTVGGPFELVDQNGAPTSEKALKGKWSAVFFGFTYCPDVCPGTLQGLAAATDQLGPKAKDFQIVFISIDP
Sco2 C. vibrioides  --------------GPFQLTDMNGKPVTEKSLLGKPTAVFFGFTYCPEVCPTTLTEMTAWLKALGKDADKLNVVLITVDP
SenC R. capsulatus  ------------IGGPFTLVDQEGRTVTDREVLAKPSLVYFGYTFCPDVCPFDMARNAQAVDILTEWGIEVTPVFISIDP
PrrC R. sphaeroides ACRKGTGSASAQIGGPFTLISETGATVTDRDVITKPSLVYFGYSYCPDVCPIDSTRNAAAVDLLAERGHDVTPVFISVDA
                                  *** * .  * ..::: :  * : *:**:::**:***      :   . *   . ..  *:*::*.

Sco1 C. vibrioides  ARDTVKQMKAYLSA--PYVPKATIGLTGTQAQVDAAAKAYRVYHAKV--GDGVDY-TMDHSTAIYLMDPKGRFKTVIPYN
Sco2 C. vibrioides  ERDTPAQLKEYLSNFDPRIQ----GFTGTPDAIAKTARAYRVYYQKVPLDGG-GY-TIDHSSAIYLFDAKGRF--VSPIA
SenC R. capsulatus  KRDTPEQLKFFAEAIHPDTI----ALTGTEAQVKAASQAYKTFY-RVQESDD-DYYLIDHSTFTYFMLPGTGF--VDFF-
PrrC R. sphaeroides ARDTPPVLTEFTDLMSPKMI----GLTGTPEQIDAAVKAYRAYYLIRNPGDP-AT-LVDHSTQTYLMDPKLGF--LDFYD
                     ***   :. : .   *       .:***   :  : :**:.::     ..      :***:  *:: .   *  :

Sco1 C. cvibrioides L--P------------PDE--IA--RR-IKDAVREG---------
Sco2 C. vibrioides  YQ-A------------PQD--RA-LGQ-LRDLLK-----------
SenC R. capsulatus  KREDTPEQIAERISCFANDSHVSTSFDARAQKSYQASRGKQMGDNHG
PrrC R. sphaeroides RDA--TPEMVADSVGCFLDA---LQTPG-DTPAA-----GNGN----
                                      :
```

**B** **C**

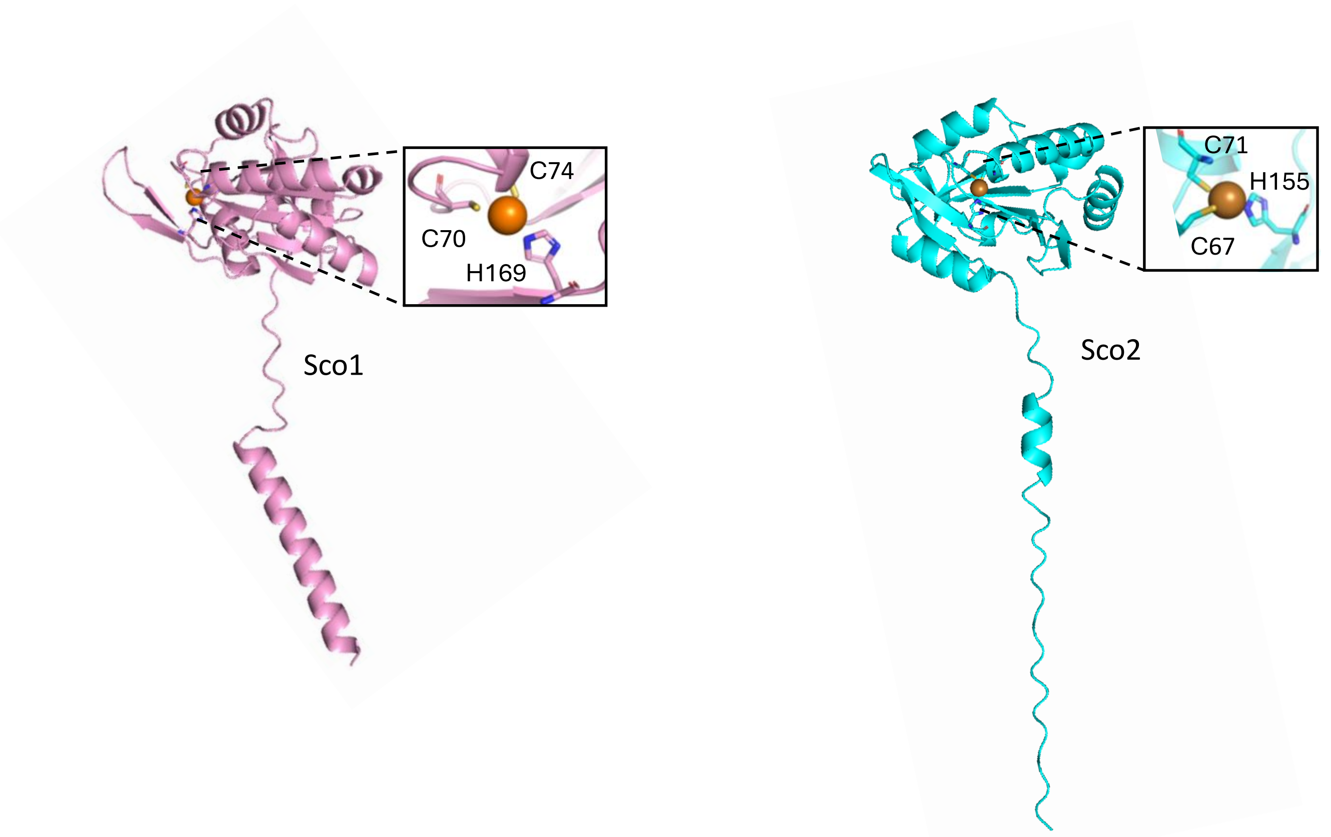


**Figure S8.** ***C. vibrioides*** **possesses two paralogs of the Sco family Cu chaperone. A.** Amino acid sequence alignment of Sco1 and Sco2 from *C. vibrioides* with its homologs SenC from *R. capsulatus* and PrrC from *R. sphaeroides*. The canonical CXXXCP motif, which is proposed to bind Cu, is highlighted in light orange in addition to the conserved H residue. **B, C.** Alphafold prediction of Sco1 and Sco2 3D structure, respectively, highlighting the conserved thioredoxin-like fold. The conserved key residues possibly involved in Cu binding are C70, C74, and H169 for Sco1 and Cys 67, Cys 71, and His 155 for Sco2

# Figure S9

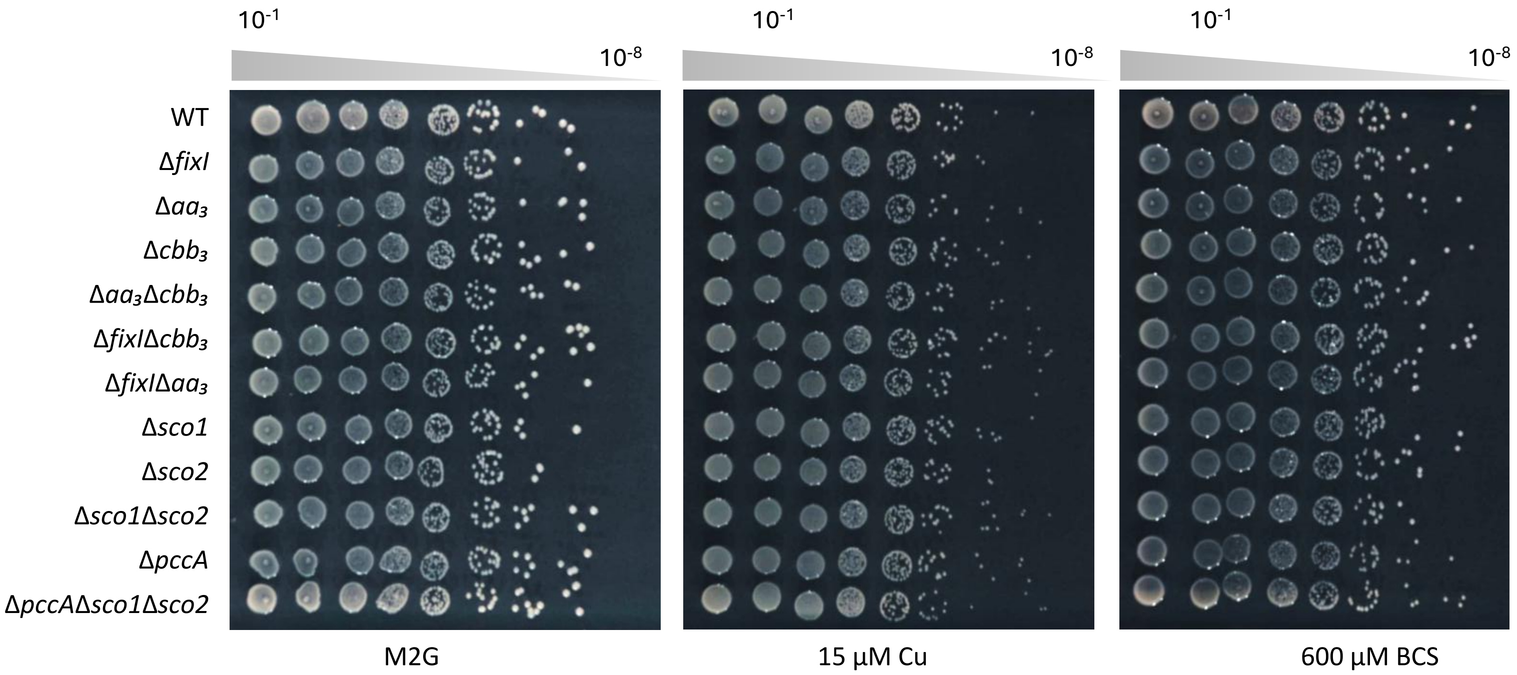


**Figure S9. The potential PccA, Sco1, and Sco2 Cu chaperones are not required for Cu resistance in C. vibrioides.** Viability assays were performed on minimal medium (M2G) under control conditions, Cu-excess (15 µM $CuSO_4$), and Cu-deficiency (600 µM BCS). Plates were incubated for 48 h at 30 °C before imaging. Biological replicates (n=3).

# Figure S10

**A**

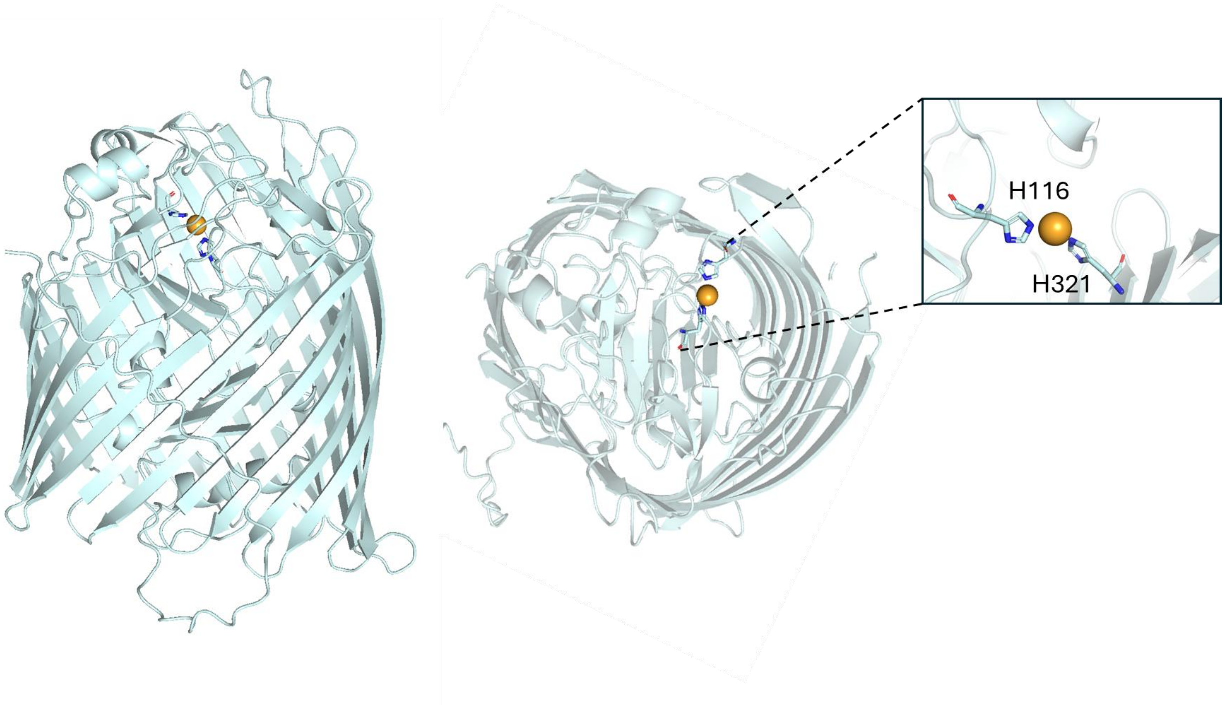


**B**

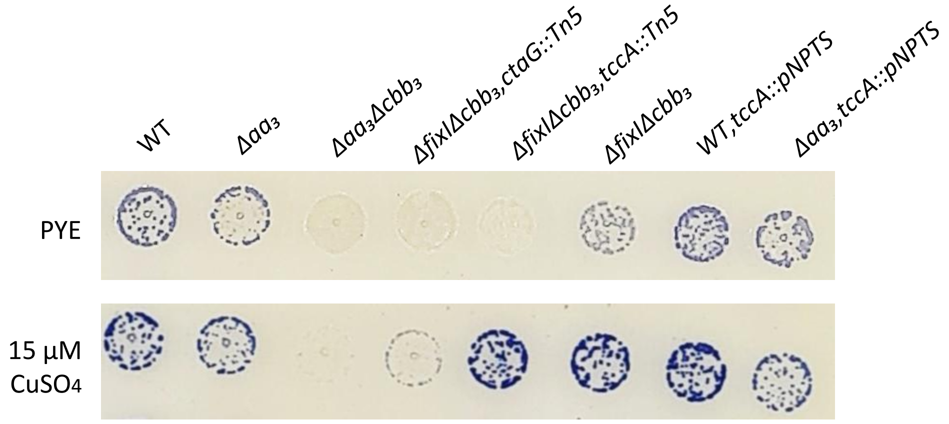


**Figure S10. TccA has a potential Cu binding site and is specifically required for *aa*$_3$-Cox. A.** Structure prediction of TccA by AlphaFold with a proposed Cu binding site composed of two His residues (H116 and H321. **B.** Growth and NADI phenotypes of colonies of *C. vibrioides* WT and mutant strains. Cells were grown aerobically at 30°C on a PYE medium and PYE supplemented with 15 µM $CuSO_4$ , and the presence of Cox activity was visualized by NADI staining. Colonies that contain WT levels of Cox activity turn dark blue within a few seconds ($NADI^+$), while those that have low or no Cox activity show lighter blue ($NADI^{slow}$) or no blue staining ($NADI^-$) upon longer exposure, respectively. (entire plate in Fig. S12B)

Figure S11

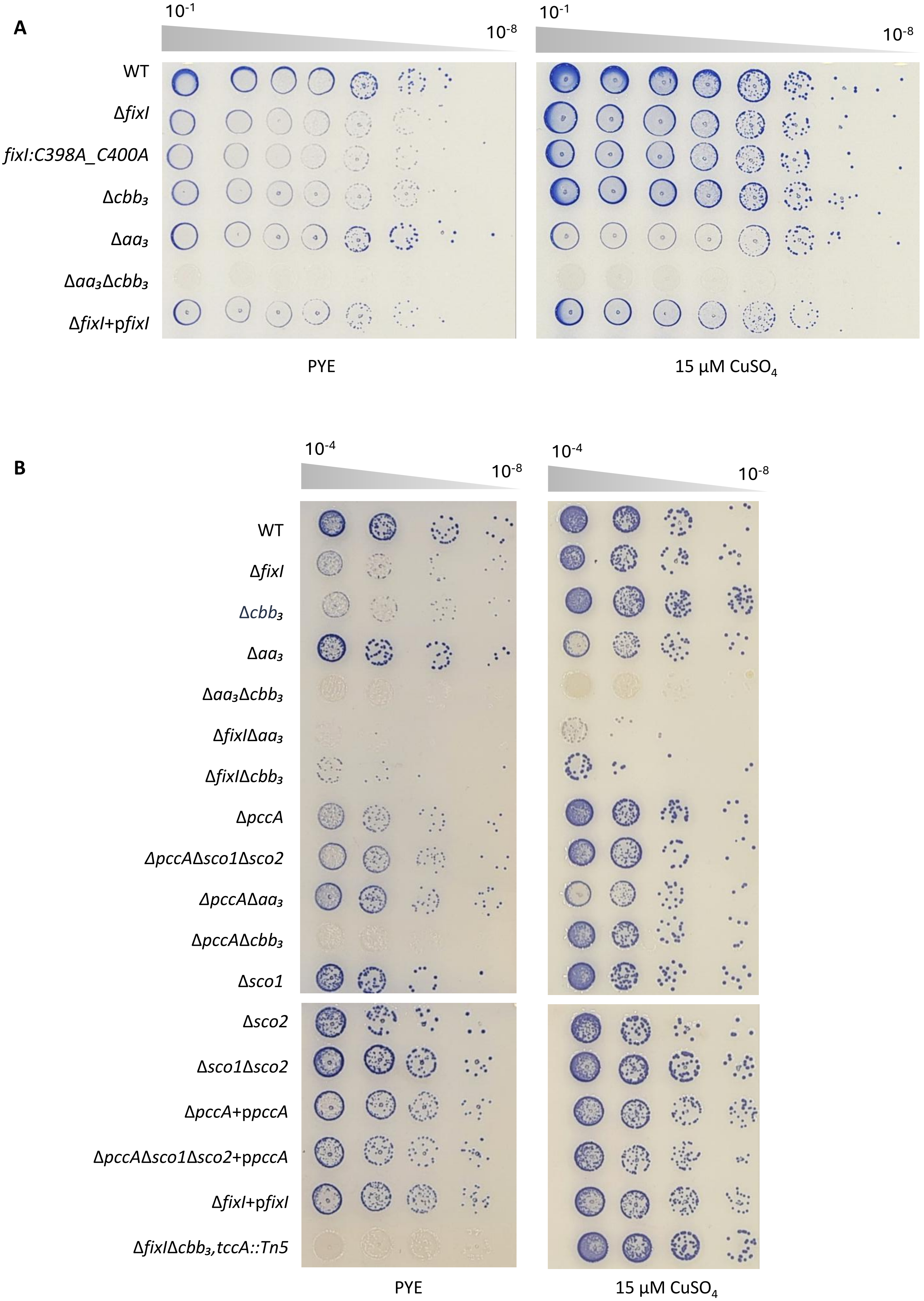


**Figure S11. Cox activity of the different *C. vibrioides* strains used in this study visualized by NADI stain in control rich medium and in the presence of 15 µM $CuSO_4$**

# Figure S12

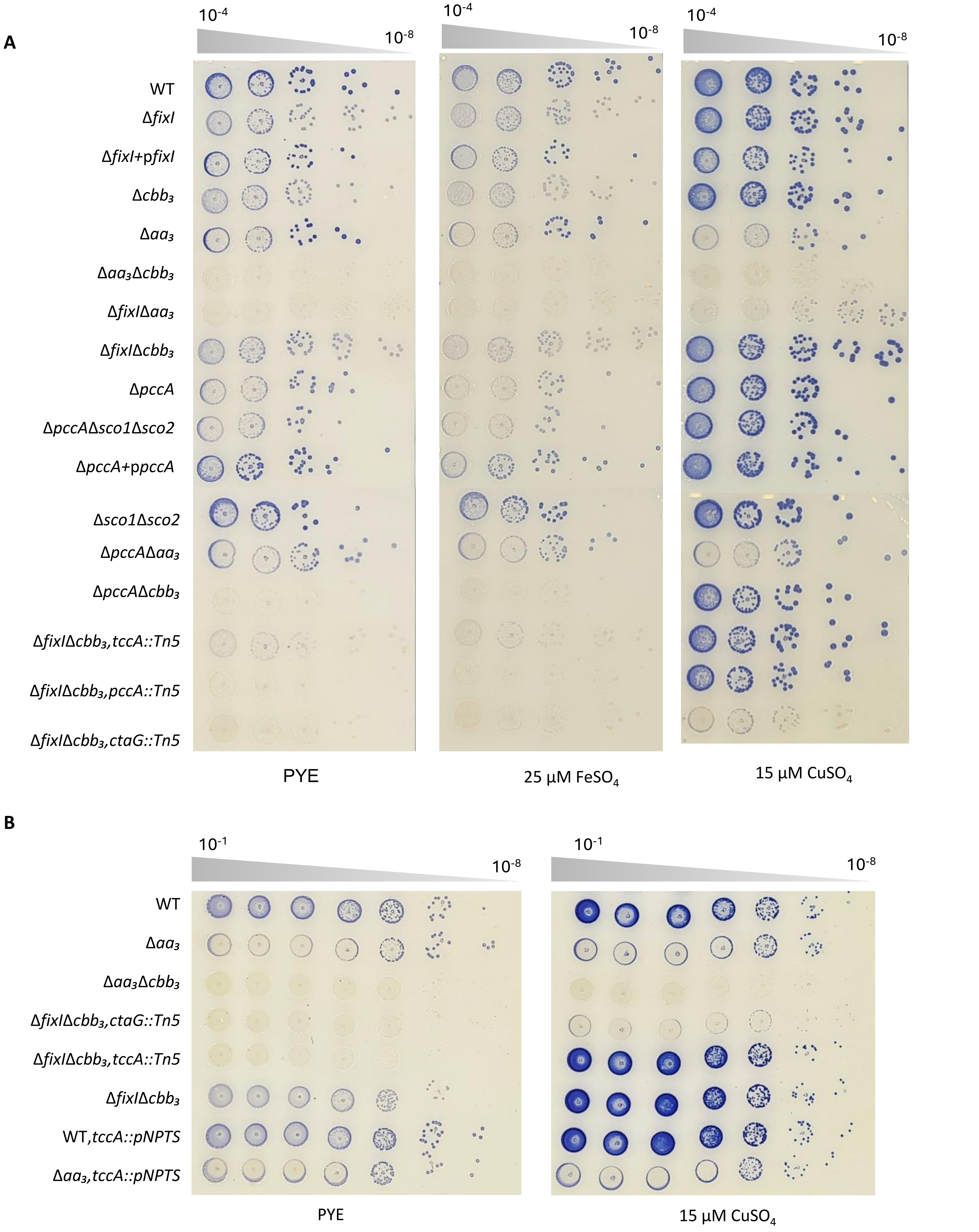


**Figure S12. Cox activity of the different *C. vibrioides* strains used in this study visualized by NADI stain in control rich medium and in the presence of 15 µM $CuSO_4$ and/or 25 µM $FeSO_4$.**
